# A prospective study of brain natriuretic peptide levels in three subgroups: Stroke with hypertension, stroke without hypertension, and hypertension alone

**DOI:** 10.4103/0972-2327.61277

**Published:** 2010

**Authors:** Zeynep Cakir, Ayhan Saritas, Mucahit Emet, Sahin Aslan, Ayhan Akoz, Fuat Gundogdu

**Affiliations:** Department of Emergency Medicine Atatürk University Faculty of Medicine, Erzurum, Turkey; 1 Department of Cardiology, Atatürk University Faculty of Medicine, Erzurum, Turkey

**Keywords:** Brain natriuretic peptide, echocardiography, hypertension, stroke

## Abstract

**Aim:**

To study brain natriuretic peptide (BNP) levels in three subgroups: patients having stroke with hypertension (HT), those having stroke without HT, and those with HT alone. We also tried to identify whether BNP levels predict the length of stay in hospital and mortality.

**Materials and Methods:**

The groups were formed by patients who had been admitted to the emergency department in the first 4–12 h after the onset of symptoms. There were 30 stroke patients with a history of HT (group I), 30 stroke patients without a history of HT (group II), and 20 HT patients without stroke (group III). Patients with congestive heart failure, chronic cor pulmonale, severe valvular heart disease, chronic renal failure, liver insufficiency, diabetes mellitus, atrial fibrillation, and those with a history of stroke were excluded from the study since these diseases can affect the plasma BNP levels.

**Results:**

The demographic characteristics, except the age distribution, were similar among the groups. The mean BNP levels in the three groups were 168.8 ± 223.9 pg/ml, 85.0 ± 75.1 pg/ml, and 84.8 ± 178.3 pg/ml, respectively. The differences between the groups were statistically significant.

**Conclusion:**

The mean BNP levels were affected by HT and/or stroke. The simultaneous presence of HT and stroke results in a more significant increase BNP than the presence of either stroke or HT alone. When diseases that can affect the plasma BNP levels are excluded, the BNP levels in stroke patients without a history of HT are similar to the levels seen in patients with only HT.

## Introduction

Stroke has been defined as a sudden onset of focal and global neurological symptoms due to diseases of cerebral blood vessels leading to hemorrhage and ischemia in the brain.[1] Besides being a serious health problem because of the high mortality and morbidity, stroke has social and economic implications. For this reason, determination of the etiology of the disease and, especially, eradication of the risk factors is of great importance.[2] Comprehensive multicenter, studies have shown that there is a positive and continuous relationship between the incidence of stroke and both systolic and diastolic blood pressures.[3,4]

Brain natriuretic peptide (BNP), which has diuretic, natriuretic, and vasodilatory effects, is a peptide-structured neurohormone released mainly from the cardiac ventricle in response to volume and pressure loads. Recent studies have shown that the plasma BNP level has an important place in the diagnosis and treatment of cardiovascular diseases, especially cardiac failure and acute coronary syndrome.[5] It is thought that BNP, produced as a result of cardiovascular changes following ischemic stroke, has an important role in the hemodynamics of these patients.[6] The BNP levels can be measured at the bedside, making it easy to follow up the patients in the emergency department (ED).[7] Several cardiac abnormalities such as myocardial necrosis and arrhythmia can develop in acute stroke patients.[8,9] These cardiac changes may cause increased BNP production by the heart[10] and, therefore, to study the relationship between BNP levels and stroke correctly, it is better to exclude those stroke patients who also have cardiac pathologies.

There have not been a sufficient number of studies in the literature comparing the plasma BNP levels between stroke patients with a history of hypertension (HT), stroke patients without a history of HT, and hypertensive patients without stroke. We therefore aimed to study BNP levels in these three subgroups: patients having stroke with hypertension, those having stroke without hypertension, and those with hypertension alone. We also tried to identify whether BNP levels could be used to predict the length of stay in hospital and mortality.

## Materials and Methods

This prospective study was performed in the emergency department of the Faculty of Medicine, Atatürk University, between November 2006 and November 2007. The subjects were patients who had been admitted to the ED in the first 4–12 h after the onset of symptoms. The three groups were as follows: 30 stroke patients with history of HT (group I), 30 stroke patients without a history of HT (group II), and 20 HT patients without stroke (group III). The body mass index (BMI) of all the patients were calculated and the neurological deficits were assessed according to the Glasgow coma scale (GCS) and National Institutes of Health Stroke Scale (NIHSS) (0–42 score). On the basis of the mean NIHSS scores, stroke patients were grouped into three according to the expected prognosis (0–6 points: good prognosis, 7–15 points: intermediate, and 16–42 points: bad prognosis).[11] Peripheral venous blood was collected in all patients and plasma BNP level was estimated by established methods: in each patient, 5 ml of blood was collected into tubes containing potassium ethylenediammine tetraacetic acid (1 mg/ml blood) by trained nurses. Within 15 min of blood collection, BNP was measured in the emergency laboratory by experienced biochemistry technicians using the Triage BNP Test (Biosite Inc., San Diego, California, USA). The precision, analytical sensitivity, and stability characteristics of the system have previously been described.[12–14] Complete blood count, routine biochemical analyses such as liver and renal function tests, brain computerized tomography (BCT), electrocardiography, and transthoracic echocardiography (TTE) were also performed. The left ventricular function was assessed by two-dimensional and Doppler echocardiography by experienced cardiologists. Standard two-dimensional images were obtained in the parasternal long and short axes, as also apical four- and two-chamber views. Informed consent forms were signed by all patients (or a close relative) before inclusion in the study. The study was approved by the ethical committee.

On the basis of the history and initial laboratory and imaging studies, patients with congestive heart failure, chronic corpulmonale, severe valvular heart disease, chronic renal failure, liver insufficiency, diabetes mellitus, atrial fibrillation (AF), and those with a history of stroke were excluded from the study (a total of 93 patients) since these diseases can affect the plasma BNP levels.

The data were analyzed using the SPSS 15 software. Data were expressed as frequencies, percentages, and means (with standard deviations). The Kruskal-Wallis test, the Mann-Whitney test, the one-way ANOVA, and the t test were used for comparison of means of continuous variables. The Chi-square test was used for analysis of the categorical variables. The Pearson and Spearman correlation test was used for assessing the correlation of BNP levels with the clinical parameters. Differences were accepted as significant when the *P*-value was < 0.05.

## Results

A total of 80 patients were included in the study. The mean age was 60.7±14.1 years (range 28–85); 47 (58.8%) of the subjects were female. BCT revealed hemorrhage in 37 patients (46.3%).The clinical features and demographic data, such as sex, age, BNP levels, and echocardiographic parameters, are presented in Table 1.

**Table 1:** Demographic and clinical characteristics in the three patient groups

	Patient groups			*P*
	
	Group I n (%) or Mean ± SD	Group II n (%) or Mean ± SD	Group III n (%) or Mean ± SD	
Age (year)	68.0±11.2	57.2±15.7	55.0±11.2	<0.05
Gender				
Female	20 (42.6)	16 (34.0)	11 (23.4)	NS
Male	10 (30.3)	14 (42.4)	9 (27.3)	
Systolic blood pressure (mm Hg)	182.1±30.9	150.8±26.5	198.5±.2	<0.001
Diastolic blood pressure (mm Hg)	106.5±18.0	91.6±14.6	115.5±14.7	<0.001
Mean arterial pressure (mm Hg)	131.6±19.9	111.3±17.0	143.2±15.3	<0.001
Blood urea nitrogen (BUN)	17.2±4.5	16.7±7.6	16.4±5.3	NS
Creatinine	0.9±0.4	0.8±0.2	0.9±0.2	NS
Brain natriuretic peptide (pg/ml)	168.8±223.9	85.0±75.1	84.8±178.3	<0.05
Left ventricular end-diastolic diameter (mm)	42.4 ± 6.1	42.3 ± 5.6	41.2 ± 5.1	NS
Left ventricular end-systolic diameter (mm)	29.9 ± 5.6	30.2 ± 6.2	27.5 ± 4.1	NS
Left ventricular ejection fraction (%)	61.4 ± 6.4	62.7 ± 7.8	64.4 ± 4.4	NS
Body mass index	26.5 ± 3.9	26.0 ± 3.5	28.3 ± 3.6	NS
Glasgow coma score (3-15 points)	12.9 ± 2.8	13.3 ± 3.1	15.0 ± 0.0	<0.001

**Figure 1:**
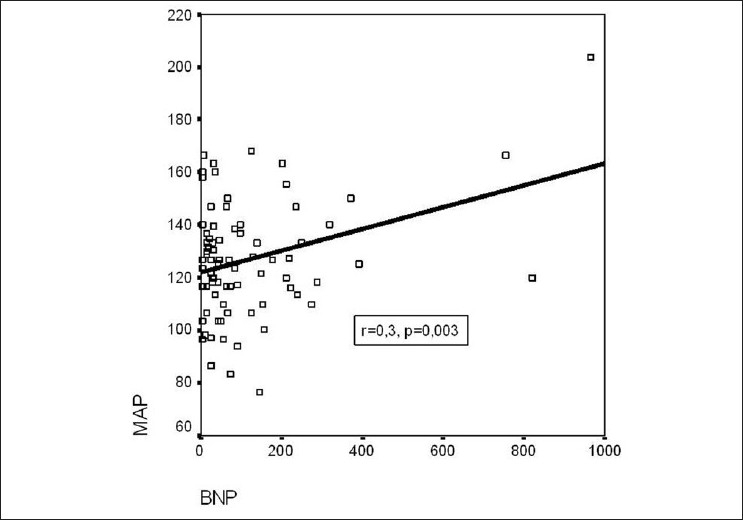
Positive correlation between MAP and BNP. MAP: mean arterial pressure, BNP: brain natriuretic peptide

**Figure 2:**
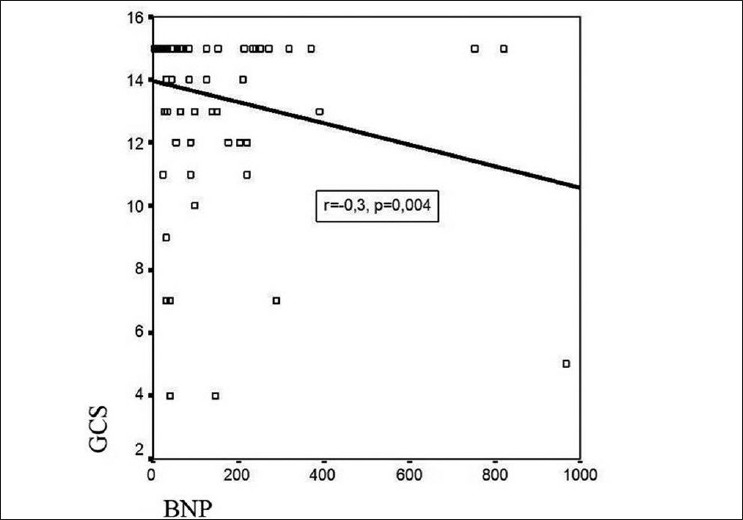
Negative correlation between GCS and BNP. GCS: Glasgow coma score; BNP: brain natriuretic peptide

The mean BNP level of all patients included in the study was 116.8±173.5 pg/ml. The mean BNP levels in groups I, II, and III were 168.8±223.9 pg/ml, 85.0±75.1 pg/ml, and 84.8±178.3 pg/ml, respectively. The differences between the groups were significant. The mean serum BNP levels in males and females were 121.03±±212.11 pg/ml and 113.92±143.79 pg/ml, respectively; this difference was not statistically significant (*P*=0.8). There was a significant positive correlation between the mean BNP levels and the ages of the patients (r = 0.53; *P*<0.001).Echocardiographic parameters of all patients were in the normal range and there were no significant differences between the groups. There was no correlation between the mean BNP levels and the left ventricular ejection fraction (LVEF) (r = -0.2; *P*=0.05), left ventricular end-diastolic diameter (LVEDD) (r = 0.2; *P*=0.1), and left ventricular end-systolic diameter (LVESD) (r = 0.1; *P*=0.3). There was a positive correlation between mean arterial pressure (MAP) and mean BNP levels (r = 0.33; *P*<0.05) [Figure. 1]. The mean score of the patients on the GCS was 13.6 ± 2.7. A significant negative correlation was found between the GCS score and the mean BNP levels (r = -0.3; *P*<0.05) [Figure. 2].

The Spearman correlation analyses for the mean BNP levels and clinical parameters of the three groups are displayed in Table 2.

**Table 2:** Correlation between BNP levels and clinical parameters in the three patient groups

	BNP					
	Group I		Group II		Group III	



	r	P	r	P	r	P

Glasgow coma score	-0.03	NS	-0.42	<0.05		
Age	0.18	NS	0.61	<0.001	0.68	<0.001
Systolic blood pressure	0.11	NS	0.27	NS	0.29	NS
Diastolic blood pressure	0.16	NS	0.15	NS	0.16	NS
Mean arterial pressure	0.1	NS	0.24	NS	0.24	NS
Blood urea nitrogen (BUN)	0.19	NS	0.49	<0.05	0.34	NS
Creatinine	0.11	NS	-0.23	NS	0.08	NS
Left ventricular end-diastolic diameter	0.27	NS	-0.15	NS	0.41	NS
Left ventricular end-systolic diameter	0.21	NS	-0.1	NS	0.14	NS
Left ventricular ejection fraction	-0.08	NS	-0.13	NS	-0.27	NS
Body mass index	0.01	NS	0.03	NS	0.42	NS

The mean duration of hypertension was 7.5±6.4 years (range 1–25 years). No correlation was found between the duration of hypertension and the mean BNP levels (*P*=0.5). The antihypertensive drugs received by the patients are listed in Table 3. Eight patients were not using any antihypertensive agent. No information was available about the drugs used in 12 patients.

**Table 3:** Antihypertensive drugs received by the patients

	Diuretic	Beta- blocker	Ca + + channel blocker	ACE inhibitor	ARB	Mix	Not received drug
Stroke patients with hypertension	1	7	2	0	0	7	2
Patients with hypertension alone	0	2	0	3	2	6	6

ACE: Angiotensin converting enzyme; ARB: angiotensin receptor blocker

Thirty-seven of the stroke patients (61.7%) had hemorrhage on BCT. There is no relationship between plasma BNP levels and hemorrhage or infarct (*P*=0.4). We studied how mean BNP levels predicted the progress of patients; the mean BNP level of stroke patients who were discharged from hospital was found to be 111.0±146.6 pg/ml and the mean BNP level of patients who died was 172.2±226.4 pg/ml; the difference was not statistically significant (*P*=0.1). Furthermore, no significant correlation was found between the length of stay in hospital and the mean BNP levels of stroke patients (r = 0.1; *P*=0.5). The mean score of stroke patients on the National Institutes of Health Stroke Scale (NIHSS) (in which the score ranges from 0–42) was found to be 10.6±6.3. There was no significant correlation between the mean NIHSS scores and the mean BNP levels (r = 0.2; *P*=0.09). There was no significant difference according to the BNP levels among the three NIHSS groups (0–6 points: good prognosis, 7–15 points: intermediate, and 16–42 points: bad prognosis) (*P*=0.2).

## Discussion

BNP, which is a member of the natriuretic peptide family, was first isolated from the pig brain in the 1980s.[15,16] Fluid-electrolyte equilibrium is maintained by the action of BNP on the central and peripheral nervous system. BNP has diuretic, natriuretic, and vasodilator effects. Diuresis and natriuresis are achieved by BNP's effect on renal hemodynamics or by a direct effect on renal tubules.[17] High plasma BNP concentrations are seen not only in patients with chronic heart failure or acute myocardial infarction but also in patients with essential HT and arrhythmias such as atrial fibrillation.[18,19] The serum level of BNP, which is a neurohormone that plays a key role in volume hemostasis, is a sensitive sign of ventricular dysfunction in symptomatic and asymptomatic patients and is closely related with the severity of dysfunction.[20–22] Excluding patients with left ventricular dysfunction and other diseases that can affect the mean BNP levels allowed us to better understand the changes in the mean BNP levels in patients with stroke and hypertension.

In the studies of Kjaer *et al*. and Wei *et al*., a negative correlation was found between LVEF and mean BNP levels.[23,24] In our study, there was a weak negative correlation between LVEF and plasma BNP levels. Decrease in the LVEF increases the tension in the ventricular wall and the mean BNP levels increase in parallel to this increase in tension. Mayer *et al*. reported a positive correlation between LVESD, LVEDD, and plasma BNP levels in a study evaluating the BNP level in patients with heart failure and coexisting mitral regurgitation.[25] In our study, no significant correlation was found between LVEDD, LVESD, and plasma BNP levels. This may be because we included patients with normal left ventricular functions to achieve a correct evaluation of stroke and mean BNP levels.

The mean BNP levels were found to be 26.2±1.8 pg/ml in patients between 55 and 64 years of age, 31.0±2.4 pg/ml in patients between 65 and 74 years of age, and 63.7±6.0 pg/ml in patients over 75 years of age.[5] The BNP levels of female patients with heart failure were higher than that of males of the same age-group.[5] In their study, Suzuki *et al*. found that there was a positive correlation between plasma BNP levels and age, regardless of whether the patient had hypertension or normotension.[26] We too found a significant positive correlation between age and plasma BNP levels in all patients. According to the literature reports there is a decrease in left ventricular compliance with age and our finding of the increase in plasma BNP levels with age is consistent with this.

Blood pressure is frequently increased in the acute phase of stroke. Deterioration in cerebral blood flow regulation, increased stress due to hospitalization, increased sympathetic activation, or increased intracranial pressure have all been blamed for this effect. Although the blood pressure was found to be high in the acute phase of stroke, HT was found only in one-third of the patients in one study.[27] In the study by Nakagawa *et al*., it was found that although patients with intracranial hemorrhage (ICH) had higher MAP levels than patients with ischemic stroke, the serum BNP levels were higher in patients with ICH than in those with ischemic stroke. In the same study, it was shown that there was a weak positive correlation between the MAP levels and the BNP levels at the beginning of the ischemic stroke.[6] In a study performed by Brosnan *et al*. in order to explore the relationship between MAP and BNP, it was found that in hypertensive patients the BNP levels increased with increase in the blood pressure.[10] However, in our study, no significant correlation was found between plasma BNP levels and MAP in any of the study groups. Nevertheless, when all the patients were evaluated carefully, it could be seen that there was a significant positive correlation between plasma BNP levels and MAP. On the basis of these results, it can be said that increase in cardiac wall tension and intracardiac pressure and volume due to increase in the MAP leads to a proportionate increase in plasma BNP level.

In the studies of Estrada *et al*. and Eguchi *et al*. it was found that BNP levels increased in the acute phase of stroke and that there was a positive correlation between blood pressure levels and BNP levels.[28,29] In the another study, on patients with subarachnoid hemorrhage, it was estimated that plasma BNP levels in patients with HT were higher than that in patients without HT.[30] In our study we found that plasma BNP levels in stroke patients with HT was higher than in stroke patients who did not have HT. This shows that HT increases the plasma BNP levels irrespective of whether stroke is present or not.

In the study of Makikallio *et al*. it was estimated that high plasma BNP levels in the acute phase of stroke were related with increased mortality and high plasma BNP levels were better prognostic indicators of mortality after stroke than the other risk factors. Furthermore, it has been reported that patients with high plasma BNP levels have a four-fold higher mortality. However, no significant correlation was noted between GCS score and BNP levels. In the study of Makikallio *et al*. it was shown that measurement of BNP in stroke patients could be used in risk classification and estimation of mortality risk.[31] In one of the studies of Sviri *et al*. it was found that there was a relationship between plasma BNP levels and the development of cerebral ischemia and neurological deficits. A strong correlation was found between GCS score and the BNP levels of the patients.[32] The NIHSS score, which is used in an ideal neurological evaluation, ranges between 0–42 points and correlates with the size of the infarct. The NIHSS score is the most important determining factor for estimation of the severity of stroke initially and for assessing the mortality risk. An NIHSS score of ≥16 is associated with higher mortality and bad functional capacity. On the other hand, an NIHSS score of ≤ 6 is related with a good functional capacity.[11] In their study, Tomita *et al*. found that there is a positive correlation between the NIHSS score and BNP levels in stroke patients.[33] However, Giannakoulas *et al*. did not find a correlation between NIHSS and BNP levels.[34] In our study, we did not find a significant correlation between NIHSS and plasma BNP levels. Moreover, although the BNP levels of the patients with NIHSS scores of ≥ 16 was greater than the levels seen in the other groups, the difference was not significant. There was a significant negative correlation between GCS score and plasma BNP levels in our study. For this reason, we thought that plasma BNP levels can be of use at the time of admission for correctly estimating the severity of the stroke, the functional capacity, and the clinical progress of stroke patients.

There are some limitations in our study. As short-term prognosis depends on many factors other than the BNP level, additional studies and more detailed statistical analyses are needed to find out the relationship of BNP with the prognosis. The size of the hemorrhage and the infarct area were not evaluated in our study. Also, the age distribution was not homogenous among the groups. All these factors may affect our conclusions. However, exclusion of systemic diseases that may have affected the BNP levels has rendered our study valuable. The mean BNP levels in our study subjects were affected only by HT and stroke. When both HT and stroke coexist there is a more significant BNP increase than when either stroke or HT alone are present. When the diseases that can affect the plasma BNP levels are excluded, plasma BNP levels in stroke patients without a history of HT are similar to the levels seen in patients with only HT.
